# A Cohort Study of the Impact of Carbapenem-Resistant *Enterobacteriaceae* Infections on Mortality of Patients Presenting with Sepsis

**DOI:** 10.1128/mSphere.00052-19

**Published:** 2019-04-10

**Authors:** Sabrina Sabino, Silvia Soares, Fabiano Ramos, Miriane Moretti, Alexandre P. Zavascki, Maria Helena Rigatto

**Affiliations:** a Infectious Diseases Service, Hospital São Lucas da PUCRS, Porto Alegre, Brazil; b Medical Sciences Post-Graduation Program, Universidade Federal do Rio Grande do Sul, Porto Alegre, Brazil; c Infectious Disease Control Service, Hospital São Lucas da PUCRS, Porto Alegre, Brazil; d Infectious Diseases Service, Hospital Moinhos de Vento, Porto Alegre, Brazil; e Intensive Care Unit, Hospital de Clínicas de Porto Alegre, Porto Alegre, Brazil; f Department of Internal Medicine, Medical School, Universidade Federal do Rio Grande do Sul, Porto Alegre, Brazil; g Infectious Diseases Service, Hospital de Clínicas de Porto Alegre, Porto Alegre, Brazil; h Pontifícia Universidade Católica do Rio Grande do Sul Medical School, Porto Alegre, Brazil; Antimicrobial Development Specialists, LLC

**Keywords:** Gram-negative bacteria, carbapenem resistant, mortality, sepsis, septic shock

## Abstract

The objective of this study is to evaluate the impact of carbapenem-resistant *Enterobacteriaceae* (CRE) infection on sepsis 30-day mortality. A retrospective cohort of patients >18 years old with sepsis and organ dysfunction or septic shock was conducted. Univariate analysis was done for variables potentially related to 30-day mortality, and the ones with *P* values of <0.05 were included in a backward stepwise hierarchic Cox regression model. Variables that remained with *P* values of <0.05 were retained in the model. A total of 1,190 sepsis episodes were analyzed. Gram-negative bacterial infections occurred in 391 (68.5%) of 571 patients with positive cultures, of which 69 (17.7%) were caused by a CRE organism. Patients with CRE infections had significantly higher 30-day mortality: 63.8% versus 33.4% (*P* < 0.01). CRE infection was also associated with a lower rate of appropriate empirical therapy (*P* < 0.01) and with the presence of septic shock (*P* < 0.01). In the hierarchic multivariate model, CRE remained significant when controlling for demographic variables, comorbidities, and infection site but lost significance when controlling for septic shock and appropriate empirical therapy. Older age (*P* < 0.01), HIV-positive status (*P* < 0.01), cirrhosis (*P* < 0.01), septic shock (*P* < 0.01), higher quick sepsis-related organ failure assessment (quick-SOFA) (*P* < 0.01), and appropriate empirical therapy (*P* = 0.01) remained in the final model. CRE infections were associated with higher crude mortality rates. A lower rate of appropriate empirical therapy and late diagnosis were more frequent in this group, and improvement of stewardship programs is needed.

**IMPORTANCE** The importance of this work relies on exploring the impact of multidrug-resistant bacterial infections such as those with carbapenem-resistant *Enterobacteriaceae* (CRE) on sepsis mortality. These infections are growing at alarming rates worldwide and are now among the most frequent and difficult-to-treat bacteria due to the very few options for susceptible antimicrobials available. This study examined 1,190 sepsis episodes, and the main findings were as follows: (i) the prevalence of CRE infections significantly increased over time, (ii) CRE infection was associated with higher 30-day mortality than that of patients with other infections (63.8% versus 33.4%), and (iii) the effect of CRE on mortality was probably influenced by the fact that those patients received lower rates of empirical therapy with active antibiotics and were also diagnosed in more advanced stages of sepsis (septic shock). Those findings point to the need for rapid diagnostic methods to identify these bacteria and the need to adjust therapeutic guidelines to this worrisome epidemiological scenario.

## Abstract (precis)

The importance of this work relies on exploring the impact of multidrug-resistant bacterial infections such as those with carbapenem-resistant *Enterobacteriaceae* (CRE) on sepsis mortality. These infections are growing at alarming rates worldwide and are now among the most frequent and difficult-to-treat bacteria due to the very few options for susceptible antimicrobials available. This study examined 1,190 sepsis episodes, and the main findings were as follows: (i) the prevalence of CRE infections significantly increased over time, (ii) CRE infection was associated with higher 30-day mortality than that of patients with other infections (63.8% versus 33.4%), and (iii) the effect of CRE on mortality was probably influenced by the fact that those patients received lower rates of empirical therapy with active antibiotics and were also diagnosed in more advanced stages of sepsis (septic shock). Those findings point to the need for rapid diagnostic methods to identify these bacteria and the need to adjust therapeutic guidelines to this worrisome epidemiological scenario.

## INTRODUCTION

Sepsis is a major public health problem, with high rates of mortality in the absence of early recognition of the syndrome and early onset of appropriate antimicrobial therapy (1). For septic patients, every hour of delay in appropriate antibiotic prescription contributes to a higher mortality risk (2).

The increasing prevalence of multidrug-resistant bacteria, in particular of carbapenemase-producing enterobacteria (or carbapenem-resistant *Enterobacteriaceae* [CRE]), has significantly restricted the therapeutic options for treatment of infections caused by these bacteria (3). This has been a particular challenge in septic patients for whom time to start appropriate antibiotic treatment is crucial for a favorable outcome (2).

Sepsis caused by CRE has been associated with lower rates of appropriate empirical therapy, which in turn is a risk factor for mortality (4). The identification of these bacteria by conventional methods takes at least 48 h, which could significantly compromise an adequate therapeutic window unless this phenotypic profile is suspected and broad-spectrum empirical therapy is implemented. Besides the therapeutic challenge, patients with multiresistant bacterial infections frequently have more comorbidities and longer hospital stays, which could also indirectly contribute to worse outcomes. Understanding the epidemiological changes that happened during recent years and the impact that they can have on mortality is an urgent step to help us build better stewardship programs and improve sepsis care (5).

In this study, we evaluate the epidemiology of CRE infections in patients with sepsis and organ dysfunction or septic shock and the impact of these infections on mortality according to septic shock status.

## RESULTS

A total of 1,336 sepsis episodes were evaluated, and 146 were excluded for occurring ≤30 days after an initial episode of sepsis or for not fulfilling organ dysfunction or septic shock criteria. We included 1,190 for analysis. Of these, 418 (35.1%) patients died within 30 days in a median time of 11 (interquartile range [IQR], 6 to 20) days after sepsis diagnosis. Patient characteristics and univariate analysis of factors associated with mortality are described in Table 1.

**TABLE 1:** Characteristics of patients and univariate analysis of risk factors for 30-day mortality in sepsis^*a*^

Variable	Total (*n* = 1,190)	30-day mortality (*n* = 418)	30-day survival (*n* = 772)	*P* value
Demographics				
Male gender, no. (%)	582 (48.9)	216 (51.7)	366 (47.4)	0.16
Age (yr), mean ± SD	63.98 ± 17.37	66.43 ± 15.47	62.4 ± 18.32	<0.01
Comorbidity, no. (%)				
Immunosuppressed (non-HIV)	175 (14.7)	71 (17.0)	104 (13.5)	0.10
HIV	55 (4.6)	28 (6.7)	27 (3.5)	0.01
Cerebrovascular disease	158 (13.3)	62 (14.8)	96 (12.4)	0.24
COPD	157 (13.2)	54 (12.9)	103 (13.3)	0.85
Cancer	259 (21.8)	111 (26.6)	148 (19.2)	<0.01
Metastatic cancer	111 (9.3)	51 (12.2)	60 (7.8)	0.01
Solid organ transplant	55 (4.6)	22 (5.3)	33 (4.3)	0.47
Diabetes	347 (29.2)	118 (28.2)	229 (29.7)	0.64
Cirrhosis	35 (2.9)	21 (5.0)	14 (1.8)	<0.01
Chronic renal disease	195 (16.4)	73 (17.5)	122 (15.8)	0.46
Hypertension	606 (50.9)	217 (51.9)	389 (50.4)	0.62
Cardiovascular disease	333 (28.0)	132 (31.6)	201 (26.0)	0.04
Charlson comorbidity index, median (IQR)	3 (1–4)	3 (1–5)	2 (1–4)	0.01
Hospital admission: >48 h from hospital admission to sepsis diagnosis, no. (%)	542 (44.7)	240 (57.4)	292 (37.8)	<0.01
Infection site or type, no. (%)				
CNS	12 (1.0)	3 (0.7)	9 (1.2)	0.55
Pulmonary	459 (38.6)	178 (42.6)	281 (36.4)	0.04
Abdominal	178 (15.0)	75 (17.9)	103 (13.3)	0.04
Urinary tract	204 (17.1)	55 (13.2)	149 (19.3)	<0.01
Skin and soft tissue	78 (6.6)	27 (6.5)	51 (6.6)	0.99
Osteoarticular	5 (0.4)	3 (0.7)	2 (0.3)	0.35
Endocarditis	7 (0.6)	3 (0.7)	4 (0.5)	0.70
Primary bacteremia	39 (3.3)	14 (3.3)	25 (3.2)	0.99
Bacteremia	298 (25.0)	136 (32.5)	162 (21.0)	<0.01
Nonidentified	22 (1.8)	10 (2.4)	12 (1.6)	0.36
Sepsis severity				
Septic shock, no. (%)	370 (31.1)	260 (62.2)	110 (14.2)	<0.01
Mechanical ventilation, no. (%)	560 (47.1)	321 (76.8)	239 (31.0)	<0.01
Quick SOFA, median (IQR)	2 (1–2)	2 (1–2)	1 (1–2)	<0.01
Bacterial or other isolate, no. (%)				
Positive culture	571 (48.0)	241 (57.7)	330 (42.7)	<0.01
Polymicrobial infection	82 (6.9)	43 (10.3)	39 (5.1)	0.01
Gram negative	391 (32.9)	171 (40.9)	220 (28.5)	<0.01
ESBL	57 (4.8)	21 (5.0)	36 (4.7)	0.78
Carbapenem-resistant nonfermentative bacteria	76 (6.4)	50 (12)	26 (3.4)	<0.01
CRE	69 (5.8)	44 (10.5)	25 (3.2)	<0.01
Gram positive	225 (18.9)	91 (21.8)	134 (17.4)	0.07
MRSA	20 (1.7)	9 (2.2)	11 (1.4)	0.35
Fungal infection	21 (1.8)	9 (2.2)	12 (1.6)	0.49
Mycobacterium tuberculosis	4 (0.3)	2 (0.5)	2 (0.3)	0.61
Therapy, no. (%)				
Appropriate empirical therapy among patients with positive cultures (*n* = 571)	260 (45.5)	83 (34.4)	157 (53.6)	<0.01
Anti-MRSA antimicrobial	97 (8.2)	45 (10.8)	52 (6.7)	0.02
Carbapenem	229 (19.2)	108 (25.8)	121 (15.7)	<0.01
Polymyxin B	59 (5.0)	27 (6.5)	32 (4.1)	0.09
Association of antibiotics	115 (9.7)	40 (9.6)	75 (9.7)	0.91

a Abbreviations: COPD, chronic obstructive pulmonary disease; CNS, central nervous system; ESBL, extended-spectrum beta-lactamase; CRE, carbapenem-resistant *Enterobacteriaceae*; MRSA, methicillin-resistant Staphylococcus aureus; IQR, interquartile range.

Positive cultures were obtained from 571 (48.0%) patients. Gram-negative bacterial infections occurred in 391 (68.5%) patients, of which 69 (17.7%) were caused by a CRE isolate: 66 (95.7%) were Klebsiella pneumoniae carbapenemase (KPC)-producing *Enterobacteriaceae*, and in 3 patients the resistance mechanism identification was not performed. The prevalence of CRE detection over the years among patients with positive cultures was as follows: 2013, 5.9%; 2014, 9.1%; 2015, 18.3%; and 2016, 15.5% (*P* = 0.03). Other resistance phenotypes such as methicillin-resistant Staphylococcus aureus (MRSA) remained stably low over the years, varying from 0% to 5% of the isolates (*P* = 0.50); extended-spectrum beta-lactamase (ESBL) producers proportionally decreased their prevalence from 17.6% in 2013 to 6% in 2016 (*P* = 0.43), and carbapenem-resistant nonfermentative Gram-negative bacteria have been detected at high rates since 2013, varying from 10.3% to 19.1% of the isolates (*P* = 0.07), none of them with statistical significance.

In 542 patients, sepsis was diagnosed within >48 h of hospital admission. Median time from hospital admission to sepsis diagnosis for CRE-infected patients was significantly higher than for patients with infections caused by other bacteria: 12 (IQR, 3.5 to 31) days versus 1 (IQR, 0 to 9.7) day, respectively (*P* < 0.01). A detailed description of patients with carbapenem-resistant infections with onset within the first 48 h of hospital admission is given elsewhere (6).

Infection sites were as follows: pulmonary tract, 459 patients (38.6%); urine, 204 (17.1%); abdominal sites, 178 (15.0%); blood, 298 (25.0%); and other sites, 51 (4.3%). CRE isolates were mostly recovered from blood (40.6%), followed by pulmonary tract (29.0%) and urine (23.2%).

Patients with CRE infections had significantly higher 30-day mortality in univariate analysis: 44 (63.8%) of 69 total versus 374 (33.4%) of 1,121 total (*P* < 0.01). They were more likely to have cancer (*P* = 0.02), bacteremia at the onset of sepsis (*P* < 0.01), and septic shock (*P* < 0.01). Compared with bacteria presenting different resistance phenotypes, CRE infections were the ones with higher 30-day mortality: CRE, 63.8%; carbapenem-resistant nonfermentative Gram-negative bacteria, 60.7%; ESBL-producing enterobacteria, 36.4%; MRSA, 35.3%; other pathogens, 31.6% (*P* < 0.01). A lower rate of appropriate empirical therapy was administered to patients with CRE infections than to patients with other identified pathogens: 9 (13.0%) of 69 total versus 250 (49.8%) of 502, respectively (*P* < 0.01). The rates of appropriate empirical therapy for CRE did not significantly change over the years (*P* = 0.40). The most common appropriate treatments given for CRE were polymyxin B in monotherapy for 5 patients; polymyxin B on combination therapy with tigecycline, amikacin, or meropenem for 3 patients; and amikacin in monotherapy for 1 patient.

**FIG 1:**
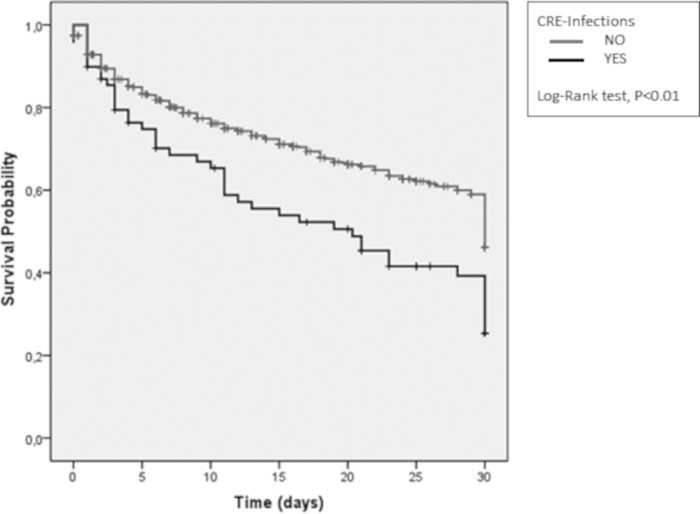
Thirty-day mortality curves for sepsis in patients with carbapenem-resistant *Enterobacteriaceae* infections versus other patients.

In survival analysis, CRE infections were significantly associated with higher 30-day mortality (*P* < 0.01) as shown in Fig. 1. CRE infections remained as an independent risk factor for 30-day mortality in the first step of the multivariate hierarchic model when controlling for age: adjusted hazard ratio (aHR), 1.70; 95% confidence interval (95% CI), 1.25 to 2.33 (*P* < 0.01). They remained significant in the second step, when comorbidities (HIV, cancer, cirrhosis, and cardiovascular disease) were added to the model: aHR, 1.58; 95% CI, 1.15 to 2.17 (*P* < 0.01). In step 3, we further controlled for infection site (bacteremia and abdominal, urinary tract, and pulmonary tract infections), and CRE persisted as an independent risk factor for mortality: aHR, 1.58; 95% CI, 1.15 to 2.18 (*P* < 0.01). In step 4, when variables associated with severity of infection (septic shock and quick sepsis-related organ failure assessment [quick SOFA]) were included in the model, the effect of CRE on mortality was no longer statistically significant: aHR, 1.20; 95% CI, 0.88 to 1.67 (*P* = 0.25). The effect of CRE remained without statistical significance with the inclusion of variables associated with therapy, such as appropriate therapy administered on the day of sepsis notification: aHR, 1.22; 95% CI, 0.88 to 1.68 (*P* = 0.23). Older age (*P* < 0.01), HIV-positive status (*P* < 0.01), cirrhosis (*P* < 0.01), septic shock (*P* < 0.01), higher quick SOFA (*P* < 0.01), and appropriate empirical therapy (*P* = 0.01) at diagnosis were independently related to 30-day mortality (Table 2).

**TABLE 2:** Hierarchic Cox regression model evaluating risk factors for mortality in sepsis patients^*a*^

Variable	HR	95% CI	*P*
Step 1, demographic			
CRE	1.70	1.25–2.33	0.01
Age (yr)	1.01	1.01–1.02	<0.01
Step 2, comorbidities			
CRE	1.58	1.15–2.17	<0.01
Age (yr)	1.01	1.01–1.02	<0.01
HIV	2.27	1.52–3.39	<0.01
Cancer	1.32	1.06–1.66	0.01
Cirrhosis	2.46	1.58–3.82	<0.01
Cardiovascular disease	1.12	0.90–1.40	0.31
Step 3, infection site			
CRE	1.58	1.15–2.18	<0.01
Age (yr)	1.02	1.01–1.02	<0.01
HIV	2.41	1.60–3.60	<0.01
Neoplasia	1.30	1.04–1.62	0.02
Cirrhosis	2.25	1.45–3.51	<0.01
Bacteremia	1.44	1.17–1.77	<0.01
Abdominal infections	1.40	1.05–1.86	0.02
Urinary tract infections	0.77	0.56–1.05	0.10
Pulmonary tract infections	1.20	0.95–1.51	0.12
Step 4, sepsis severity			
CRE	1.20	0.88–1.67	0.25
Age (yr)	1.01	1.01–1.02	<0.01
HIV	2.06	1.37–3.12	<0.01
Neoplasia	1.02	0.96–1.50	0.10
Cirrhosis	1.79	1.14–2.79	0.01
Abdominal site infection	1.14	0.88–1.47	0.32
Bacteremia	1.11	0.90–1.37	0.32
Septic shock	3.30	2.67–4.08	<0.01
Quick SOFA	1.22	1.08–1.38	<0.01
Step 5, therapy			
CRE	1.21	0.88–1.68	0.23
Age (yr)	1.01	1.01–1.02	<0.01
HIV	1.93	1.28–2.90	<0.01
Cirrhosis	1.82	1.17–2.85	<0.01
Septic shock	3.52	2.86–4.34	<0.01
Quick SOFA	1.21	1.07–1.37	<0.01
Appropriate empirical therapy	0.73	0.57–0.93	0.01

a Abbreviations: HR, hazard ratio; CI, confidence interval; CRE, carbapenem-resistant *Enterobacteriaceae*.

### Stratification according to the presence of septic shock.

Of 370 patients with septic shock, 260 (70.3%) died in 30 days compared to 158 (19.3%) of 820 patients without septic shock (*P* < 0.01). In the first group, cancer (*P* = 0.02), abdominal site infections (*P* < 0.01), polymicrobial infections (*P* = 0.01), and bacteremia (*P* < 0.01) were significantly more frequent than in other patients. The prevalence of CRE in patients with and without septic shock was 33 (8.9%) of 370 and 36 (4.4%) of 820 patients, respectively (*P* < 0.01). Results from stratified Cox regression analysis are presented in Table 3. CRE remained one of the main risk factors for mortality in patients without septic shock in multivariate analysis (*P* < 0.01), along with cirrhosis (*P* < 0.01), HIV-positive status (*P* < 0.01), older age (*P* < 0.01), and a higher quick SOFA (*P* < 0.01). In patients with septic shock, only older age (*P* < 0.01) and cirrhosis (*P* = 0.04) were independent risk factors for mortality, while appropriate empirical therapy was a protective factor (*P* = 0.01).

**TABLE 3:** Stratified Cox regression analysis according to septic shock status^*a*^

Variable	Septic shock			No septic shock		
	HR	95% CI	*P* value	HR	95% CI	*P* value
CRE	0.87	0.57–1.33	0.52	2.36	1.46–3.83	<0.01
Age	1.01	1.00–1.102	<0.01	1.01	1.01–1.03	<0.01
HIV status	1.62	0.96–2.72	0.07	2.40	1.25–4.64	<0.01
Cirrhosis	1.77	1.04–3.00	0.04	3.13	1.37–7-19	<0.01
Quick SOFA	1.06	0.91–1.24	1.06	1.43	1.17–1.74	<0.01
Appropriate empirical therapy	0.69	0.51–0.92	0.01	0.89	0.59–1.37	0.60

a Abbreviations: HR, hazard ratio; CI, confidence interval; CRE, carbapenem-resistant *Enterobacteriaceae*.

Of note, in the subgroup without septic shock, appropriate empirical therapy was administered to 1 (2.8%) of 36 patients with CRE infections compared to 169 (53.8%) of 314 patients with infections with other identified pathogens (*P* < 0.01). In septic shock patients, appropriate empirical therapy for CRE was administered to 8 (24.2%) of 33 patients versus 81 (43.1%) of 188 patients with other infections (*P* = 0.054).

## DISCUSSION

In this study, we evaluated the impact of CRE infections on 30-day mortality among 1,190 patients identified with sepsis. Although, during the cohort period, sepsis definition included all patients with at least 2 systemic inflammatory response syndrome (SIRS) criteria and presence of infection, for this study we selected only cases in which organ dysfunction or sepsis shock was documented, in accordance with the latest sepsis definitions (1). All patients were followed according to an institutional protocol and received treatment, although eventual inadequacies of empirical treatments were retrospectively identified and analyzed in this work.

Gram-negative bacteria were the most prevalent causative pathogens of sepsis and CRE infections accounted for 17.7% of these cases. While other bacteria with worrisome antimicrobial susceptibility phenotypes, such as MRSA, ESBL-producing *Enterobacteriaceae*, or carbapenem-resistant nonfermentative bacteria, remained stable over time, CRE infections showed a significant increase of prevalence in the last 2 years of analysis compared to the beginning of the cohort. The growing incidence of CRE infections in hospitalized patients has been reported worldwide (5). Our data showed a high predominance of KPC among CRE isolates, as reported in other studies (7). Time from hospital admission to sepsis diagnosis was significantly longer in patients with CRE infections than in others. We think that this may reflect either the fact that length of hospitalization can be a risk factor for acquiring CRE infections or a delay in recognizing sepsis syndrome in these patients.

CRE infections were associated with higher 30-day mortality in crude survival analysis. They showed higher absolute mortality also compared with the other different bacterial resistance phenotypes analyzed. In fact, higher mortality of infections with KPC-producing bacteria has already been shown in specific groups of patients with enterobacterial bloodstream infections, including infections with ESBL-producing bacteria (8). Differently from previous studies, we compared CRE infection mortalities among all patients identified with sepsis and organ dysfunction, regardless of the causative pathogen. Even in this more heterogeneous scenario, which accounted for different infectious agents with a broad range of resistance mechanisms, CRE infections were associated with the highest mortality rates.

In the hierarchic multivariate analysis, CRE infections remained independently related to the outcome after controlling for demographic variables, comorbidities, and infection site. Interestingly, this association was lost when controlling for septic shock and appropriateness of empirical antibiotic therapy. We offer some hypotheses to explain this finding. First, a significantly higher prevalence of CRE infections was found in patients with septic shock, showing collinearity between these variables. The fact that patients with CRE infections were more often diagnosed with septic shock may be attributed to a late diagnosis of the syndrome or even to a potential higher virulence of these bacteria (9). Second, the impact of an appropriate empirical treatment suggests that failure in recognizing patients at risk of carbapenem-resistant infections and delay in appropriate therapy may explain the worse outcomes. Other studies have also suggested that inappropriate empirical therapy in CRE infections plays a major role in the poor outcomes of patients with these infections (4, 10).

In our final model, older age, HIV infection, cirrhosis, septic shock, higher quick SOFA, and empirical antibiotic susceptibility remained independent risk factors for mortality. All these variables have been previously associated with sepsis mortality (10–12).

We did a stratified analysis for the presence of septic shock. When CRE infections were diagnosed in patients with septic shock, the rate of appropriate empirical therapy was higher than when they were diagnosed in patients without shock. It is reasonable to think that in more clinically unstable patients, physicians have decided to prescribe broader-spectrum antibiotics as first-line regimes, which may have reduced the impact of this resistance phenotype on mortality. Although appropriateness of antibiotic therapy was proportionally higher in patients with septic shock, hemodynamic instability led to a more-than-3-times-higher overall mortality. This finding clearly shows that early diagnosis of this syndrome (before onset of septic shock) is the main goal of sepsis treatment.

Our study has some limitations. This is a single-center analysis, and data must be critically interpreted as they may not be relevant for different epidemiological scenarios where CRE infections are not as prevalent. Moreover, the exact time from the onset of sepsis symptoms, diagnosis, antibiotic administration, and completion of sepsis bundle could not be estimated. We understand that these could impact the outcome even in cases where appropriate antibiotic treatment was ensured (2). Last, we chose to analyze the whole cohort of patients identified with sepsis and organ dysfunction, although we could not recover the microbiological causative agent in most of them. We think that presenting data in this perspective shows a more accurate scenario of what we face in real-life clinical practice and the crude impact of CRE infections on sepsis mortality.

This study provides us an important awareness about the growing incidence of CRE infections. This fast epidemiological shift may explain the challenge in appropriateness of protocols for empirical treatment of patients. We know from other studies that the consequences go beyond clinical aspects and also hugely impact total health costs and length of hospital stay (13). The implementation of rapid tests for carbapenemase detection could be a future direction for guiding therapy and needs to be better evaluated in this scenario (14). Meanwhile, we need to consider the possibility of Gram-negative bacterial resistance when deciding on empirical treatment in patients with severe infections—especially in settings such as ours where Gram-negative bacteria are very prevalent pathogens. This must be balanced with the risk of using unnecessary broad-spectrum antibiotics and contributing to even worsening our epidemiological scenario. Constant vigilance and updates of stewardship practice are necessary for adequate treatment of these patients and reduction of sepsis mortality rates.

## MATERIALS AND METHODS

### Study design, settings, and participants.

We conducted a retrospective cohort study from November 2013 to May 2016, in a 593-bed tertiary care hospital in Porto Alegre, Brazil. We included patients ≥18 years old who had sepsis diagnosis and at least one organ dysfunction or septic shock.

In the beginning of 2013, a sepsis bundle adapted from the Surviving Sepsis Campaign (2013) protocol (15) was implemented in our hospital, and nurses and medical staff were trained for early sepsis diagnosis and management.

### Sepsis protocol.

All patients at emergency arrival or during hospitalization were screened for the following criteria: temperature of ≥38°C or <36°C, cardiac frequency of >90 rpm, respiratory frequency of >20/min, systolic blood pressure of <90 mm Hg, median blood pressure of <65, mental confusion, chills, headache with stiff neck, total white blood cell level of >12,000 or <4,000 ml/dl, and >10% young forms in the blood count. Whenever two criteria were fulfilled, the patient was assigned to the sepsis control program. Additional exams and evaluation for organ dysfunction were done with blood cultures, arterial and venous blood gases, blood count, creatinine, platelets, lactate, bilirubin, and partial thromboplastin. Patients were considered to have a second sepsis episode if it occurred more than 30 days after the first episode and a different pathogen (or infection site) was documented.

This study was approved by the local ethics committee.

### Variables and definitions.

Primary outcome was 30-day mortality after sepsis diagnosis. Variables possibly related to 30-day mortality were analyzed: demographics (age and gender), comorbidities, Charlson comorbidity index, severity of the disease (quick SOFA [1]), need for vasopressors or mechanical ventilation, infection site, bacterial isolates and antimicrobial susceptibility profile, and antibiotic regimen prescribed at the time of diagnosis.

### Microbiology.

Bacterial identification and antimicrobial susceptibility tests were performed using the Vitek 2 (bioMérieux, France) automated system. Susceptibility was interpreted according to Clinical and Laboratory Standards Institute (CLSI) guidelines. KPC identification was done by phenotypic test, followed by multiplex real-time PCR. Appropriate empirical treatment was defined as receiving at least one antimicrobial with *in vitro* activity against the isolate on the day of sepsis diagnosis. Coagulase-negative *Staphylococcus* isolates recovered in only one blood sample were considered contaminants, as well as *Candida* species isolated in urine cultures.

### Statistical analysis.

We used SPSS for Windows, version 18, for statistical analysis. Univariate analyses were performed using the Fisher exact test for categorical variables and the Student *t* test or Mann-Whitney U test for continuous variables. All tests were two-tailed, and a *P* value of <0.05 was considered statistically significant.

A hierarchic Cox regression model (16) was used, including first demographic variables, followed by comorbidities, infection site, sepsis severity, and therapy. Variables with *P ≤ *0.05 in univariate analysis were included in each step in a backward stepwise model. Those with a *P* value of *≤ *0.05 were maintained in the final model. CRE was maintained in the model regardless of the *P* value because it was our main testing variable. Variables were checked for confounding and collinearity.

We also performed a stratified analysis for patients with and without septic shock.
